# Disrupted adipokine secretion and inflammatory responses in human adipocyte hypertrophy

**DOI:** 10.1080/21623945.2025.2485927

**Published:** 2025-04-03

**Authors:** Dan Gao, Chen Bing, Helen R. Griffiths

**Affiliations:** a Institute of Molecular and Translational Medicine, School of Basic Medical Sciences, Xi’an Jiaotong University Health Science Center, Xi’an, China; b Ministry of Education, Key Laboratory of Environment and Genes Related to Diseases Xi’an Jiaotong University, Xi’an, China; c Department of Human Anatomy, Histology and Embryology, School of Basic Medical Sciences, Xi’an Jiaotong University Health Center, Xi’an, China; d Institute of Life Course and Medical Sciences, University of Liverpool, Liverpool, UK; e Swansea University Medical School, Swansea University, Swansea, UK

**Keywords:** Obesity, human adipocyte, hypertrophy, adipokines, secretion

## Abstract

Adipocyte hypertrophy is a critical contributor to obesity-induced inflammation and insulin resistance. This study employed a human adipocyte hypertrophy model to investigate the adipokine release, inflammatory responses, and the intracellular singling pathways. Hypertrophic adipocytes exhibited increased lipid content and lipolysis, a decline of anti-inflammatory adipokine adiponectin release and an increase of pro-inflammatory adipokine leptin release compared to mature adipocytes. Moreover, TNFα and LPS exacerbated the decrease in adiponectin secretion by hypertrophic adipocytes while promoting the secretion of leptin, MCP-1 and IL-6, which is associated with impaired activation of p38 and JNK MAPK and persistent activation of ERK and IκBα in hypertrophic adipocytes. These altered adipokine secretions and inflammatory responses within hypertrophic adipocytes may contribute to adipocyte dysfunction in human obesity.

## 1. Introduction

Obesity has emerged as a significant global health challenge and associated with many diseases such as type 2 diabetes, non-alcoholic liver diseases, and certain types of cancer [1]. Obesity-related alterations in adipose tissue are characterized by an enlargement of adipose tissue and accompanied with a series metabolic dysfunction such as inflammation and insulin resistance [2]. There are two ways of adipose tissue enlargement: hyperplasia and hypertrophy. Adipocyte hypertrophy is intricately linked to the development of metabolic complications such as inflammation and insulin resistance [2]. Understanding the molecular mechanisms underlying adipocyte hypertrophy is crucial for elucidating the pathophysiology of obesity-related disorders.

Adipocytes, which function as an energy storage system, can also respond to various physiological signals or metabolic stresses by secreting endocrine factors that regulate a variety of processes, including appetite regulation, glucose balance, insulin sensitivity, inflammation, and tissue healing [3]. Altered adipokine secretion by adipocytes is linked to chronic inflammation and obesity-related metabolic disorders [4–6]. Moreover, *in vitro* chronological culture of mouse 3T3-L1 adipocytes are characterized by a decrease in adipogenic abilities and altered expressions of proinflammatory cytokines [7,8]. Nevertheless, little is known about the metabolism and the secretory pattern of adipokines in human adipocytes with hypertrophy.

In this study, we employed an *in vitro* human adipocyte hypertrophy model to examine the adipokine release profiles, inflammatory responses, and intracellular signalling pathways in hypertrophic adipocytes compared to their mature counterparts. Our findings indicate that human hypertrophic adipocytes are characterized as a considerable increase in lipid content and lipolysis, a reduced anti-inflammatory adipokine adiponectin and increased pro-inflammatory adipokine profiles. Moreover, inflammatory stimuli LPS and TNFα significantly exacerbated the decrease in adiponectin secretion by hypertrophic adipocytes while promoting the secretion of pro-inflammatory adipokine leptin and cytokine MCP-1 and IL-6. Finally, we found an altered intracellular singling in hypertrophic human adipocytes in response to acute TNFα treatment with impaired activation of p38 and JNK MAPK and persistent activation of ERK and IκBα compared to mature adipocytes. Collectively, these findings indicate that altered adipokine secretions and inflammatory responses in human hypertrophic adipocytes may contribute to adipocyte dysfunction in individuals with obesity.

## 2. Materials and methods

### 2.1. Human primary preadipocytes culture and differentiation

Human primary preadipocytes were obtained from subcutaneous adipose tissue of a female Caucasian subject (body mass index 21 kg/m^2^, age 44 years) and purchased from PromoCell Product No. C-12730; Heidelberg, Germany). The preadipocytes with passage number less than 10 were used and cultured in a preadipocytes growth medium at 37°C in a 5% CO2/95% air as previously described [9,10]. Upon confluence, the cells were incubated for 3 days in differentiation medium (Dulbecco’s modified Eagle’s medium-Ham’s F-12 (1:1) medium (Gibco BRL, GFigrand Island, NY) containing 32 μM biotin (Sigma), 1 μM dexamethasone (Sigma), 200 μM 3-isobutyl-1-methylxanthine (Sigma),100 nM insulin (Sigma), 11 nM L-thyroxine 8 μM rosiglitazone (Sigma), 100 U/mL penicillin, and 100 μg/mL streptomycin) and followed by maintenance medium (DMEM/f12 supplementted with 3% foetal bovine serum (Biological Industries, Israel), 100 nM insulin, 32 μM biotin, and 1 μM dexamethasone) until full differentiation into adipocytes. The maintenance medium were changed every 2 days. Culture media were collected at various time points (D0-D30) throughout the differentiation process.

### 2.2. Cell treatment

In order to investigate the impact of LPS (Sigma) and TNF-α (Sigma) on the secretion of adipokines, human adipocytes at various stages of differentiation (day 12, day 18, and day 24) were exposed to LPS (5 or 100 ng/ml) and TNF-α (5 or 25 ng/ml) for 48 h.

### 2.3. Oil Red O and intracellular triglyceride (TAG) measurement

Lipid storage in adipocytes was assessed using Oil Red O staining. Adipocytes were fixed with 10% formalin for 30 min at room temperature on days 6, 12, 18, 24, and 30 post-differentiation initiation. Subsequently, the cells were stained with a 0.3% Oil Red O solution (Sigma) for 1 h at room temperature. Following three washes with PBS, the red-stained lipid droplets were observed and captured under a light microscope. Intracellular triglyceride (TG) content was quantified using a TG kit (Sigma).

### 2.4. Glycerol release assay

Basal lipolysis was assessed by quantifying glycerol release in the cell culture medium through a colorimetric assay a previous described [10]. In brief, the cell culture medium (25 μl) or various dilutions of a glycerol standard solution (Sigma) were combined with a free glycerol reagent (200 μl, Sigma) and incubated at room temperature for 10 min. The absorbance of the samples and standard were measured using a spectrophotometer (Bio-Rad) at a wavelength of 540 nm. The concentration of glycerol was then determined by referencing a glycerol standard curve.

### 2.5. Multiplex ELISA

The levels of adiponectin, leptin, IL-6, IL-8, and MCP1 proteins in the culture medium of adipocytes were quantified using the Bio-Plex cytokine assay (Bio-Rad, UK). Specifically, the medium was incubated with fluorescently labelled beads that were conjugated with monoclonal antibodies specific to leptin, IL-6, IL-8, and MCP1 (Bio-Rad, Hercules, USA). The bead-sample conjugate was then incubated with a biotinylated secondary detection antibody and a streptavidin-PE fluorophore. Subsequently, the samples were analysed using a Luminex-200 platform with Bioplex software version 5 (Bio-Rad, Hercules, USA).

### 2.6. ELISA

The protein concentrations of adiponectin, leptin, MCP1, and IL-6 secreted by adipocytes following treatment with TNFα or LPS were assessed using ELISA kits (R&D Systems, Abingdon, UK) to measure the levels in the cell culture medium.

### 2.7. Western blotting

Total cellular protein was extracted from human adipocytes following a previously established protocol [10]. Briefly, human adipocytes were washed three times with cold PBS and lysed in lysis buffer (containing 50 mm Tris-HCl pH 6.7, 10% glycerol, 4% SDS, 2% 2-mercaptoethanol) on ice for 30 min. After homogenization by pipetting, the protein concentrations were determined by BCA protein assay (Thermo Scientific, Rockford, IL). Protein samples (20 μg/lane) were separated on 10% SDS polyacrylamide gels and transferred onto a nitrocellulose membrane (Millipore) by wet transfer (Trans Blot; Bio-Rad) at 300 mA for 90 min. The membrane was blocked at room temperature for 1 h with Tris-buffered saline (TBS) containing 0.1% Tween-20 and 5% bovine serum albumin (BSA). It was then incubated overnight at 4°C with the antibody against p-p38, p-JNK, p-ERK and IκBα (1:1000 dilution, Cell Signaling Technology, Beverley, MA). Following this, the membranes were incubated with secondary antibodies (1:2000 dilution, Jackson Immunoresearch, West Grove, PA) at room temperature, washed three times with TBS-0.1% Tween-20, and then exposed to a chemiluminescence substrate (Bio-Rad, Hercules, CA) for signal detection using a Molecular Imager ChemiDoc XRS+ System (Bio-Rad). Finally, the membrane was reprobed with α-tubulin (1:1000 dilutions, Santa Cruz Biotechnology) as a loading control.

### 2.8. Statistical analysis

The data were presented as means with standard error. The comparison among multiple groups was performed using one-way ANOVA followed by Bonferroni’s t-test. Statistical significance was determined when *p* < 0.05.

## 3. Results

### 3.1. Establishing an in vitro model of human adipocyte hypertrophy

**Figure 1.**
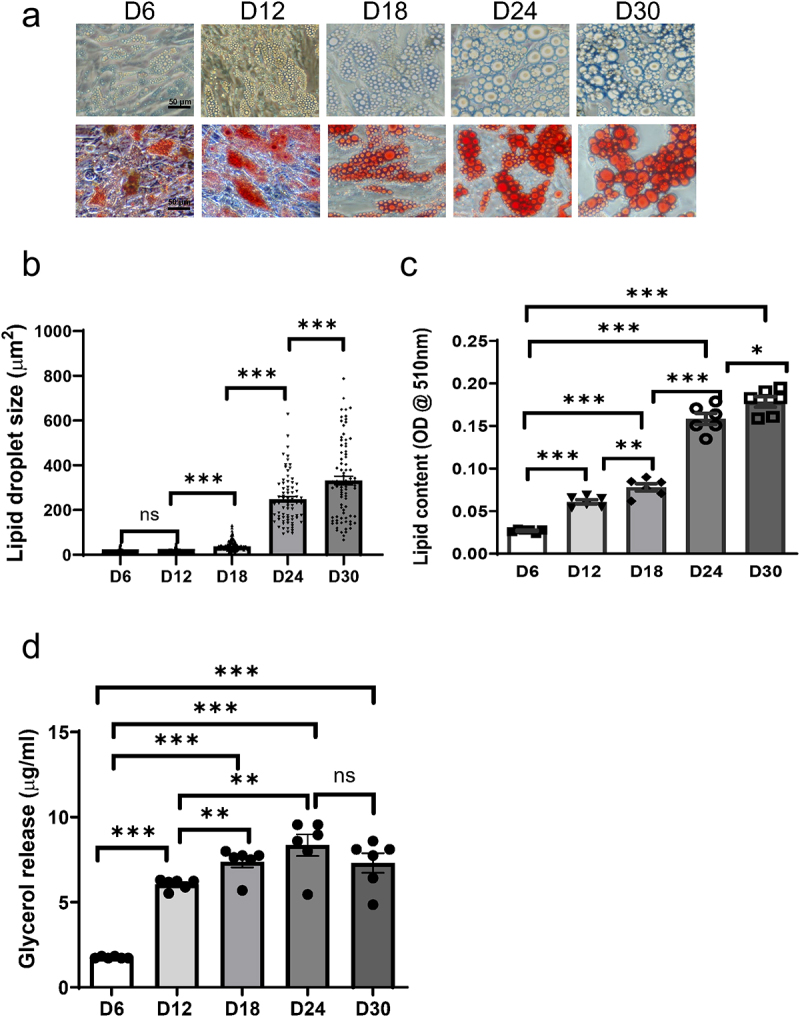
Lipids accumulation and glycerol release from human primary preadipocytes at post-differentiation day 6 to day 30. Preadipocytes were differentiated and incubated for up to 30 days. (a) Lipids accumulation by phase contrast microscopy and Oil Red O staining. (b) Lipid droplet size were determined by image J. (c) Lipid contents were determined by colorimetric measurement at 550 nm. (d) Glycerol release. Results are expressed as means ± SEM (bars) for groups of 6. *p < .05, **p < .01,***p < .001.

Since the *in vitro* differentiation of preadipocyte from obese humans are limited [11], we therefore cultured human preadipocytes from lean subject and differentiate into mature human adipocytes (D9-D12) and hypertrophic adipocytes with long-term culture (D24-D30). Initially, we examined the lipid accumulation and lipolysis patterns subsequent to the differentiation of human adipocytes. We captured phase contrast images of human preadipocytes at various time points post-differentiation, ranging from D6 to D30, and noted an augmentation in the size of lipid droplets at D12, D18, D24, and D30 compared to D6 (Figure 1a upper panel). Additionally, to quantitatively assess the lipid content in adipocytes, we performed Oil Red O staining and quantification of lipid content. The results showed a notable rise in lipid droplet size (Figure 1b) and lipids contents (Figure 1a lower panel, Figure 1c) in adipocytes at D12 post-differentiation, with further increases at D18, D24, and D30, culminating in an almost threefold elevation at D30 (Figure 1c). Furthermore, glycerol release exhibited a substantial increase at D12, reaching a plateau after D18 (Figure 1d). In addition, we assessed the insulin sensitivity of hypertrophic adipocytes by measuring the insulin-stimulated Akt phosphorylation at Ser473. As shown in Fig S3, compared with adipocytes at day 12, those at day 24 exhibited a significant decrease in Akt phosphorylation at Ser473 upon insulin stimulation. This finding indicates that adipocytes with hypertrophy are insulin resistant.

### 3.2. Basal adipokine secretion profiles of human adipocytes in culture

**Figure 2.**
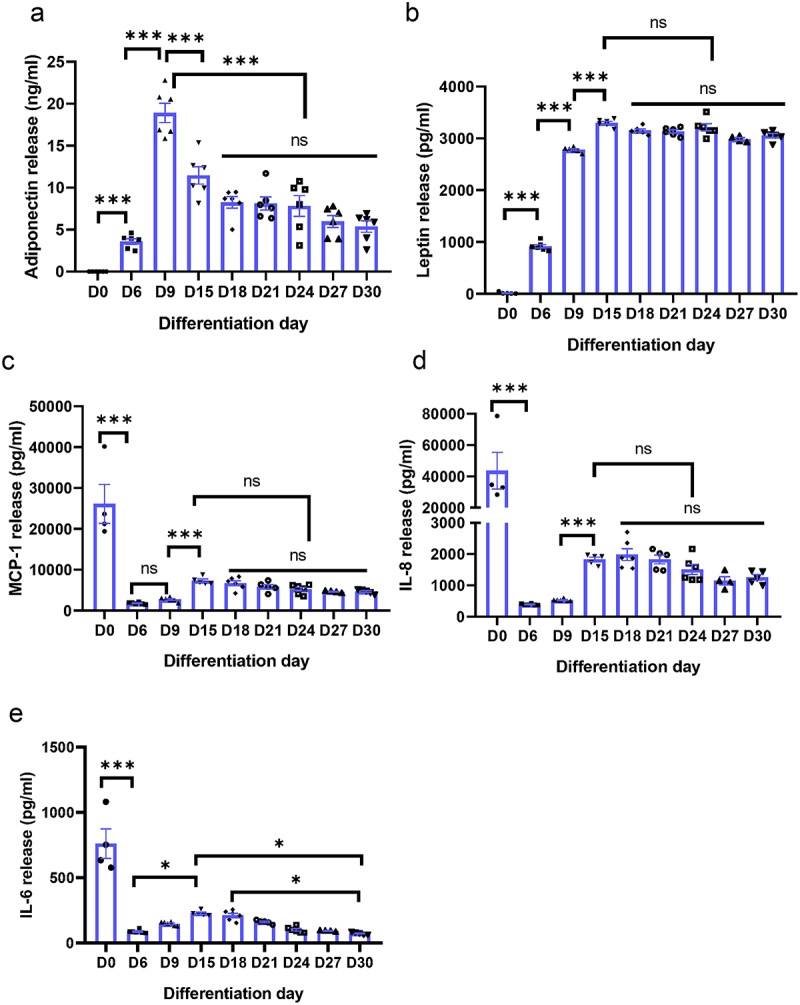
Adipokine and inflammatory cytokine release from human primary preadipocytes (D0) and post-differentiation up to day 30 (D30). Preadipocytes were differentiated and incubated for up to 30 days. Levels of adipokine adiponectin (a), leptin (b) and inflammatory cytokine MCP-1 (c), IL-8(d) and IL-6 (e) release into cell culture medium were measured by ELISA. Results are expressed as means ± SEM (bars) for groups of 6. *p < .05, **p < .001.

Next, we investigated the basal release profiles of adipokines of adipocytes in culture. A multiplex cytokine ELISA was employed to analyse the adipokines secretion from preadipocytes (D0) to hypertrophic adipocytes (D30). The results showed that the basal secretion of the anti-inflammatory adipokine adiponectin, was nearly undetectable in preadipocytes (D0), but peaked at D9 and subsequently declined (Figure 2a). Conversely, the release of pro-inflammatory cytokine leptin reached its peak at D15 and remained consistent thereafter (Figure 2b). In contrast, cytokines including MCP-1, IL-8, and IL-6 exhibited significantly higher levels in preadipocytes (D0) and markedly decreased at D6 post differentiation (Figure 2c–e). Similar to leptin, these cytokines also peaked at D15 and maintained their levels thereafter (Figure 2c–e). Notably, only the secretion of adiponectin (Figure 2a) and IL-6 (Figure 2e) in hypertrophic adipocytes exhibited differences when compared to mature adipocytes (D9 or D15). This implies that, under basal conditions, there is a relatively mild alteration in hypertrophic adipocytes compared to mature adipocytes (D9 - D15).

### 3.3. Unbalanced adipokine secretion in LPS and TNFα treated hypertrophic human adipocytes

**Figure 3.**
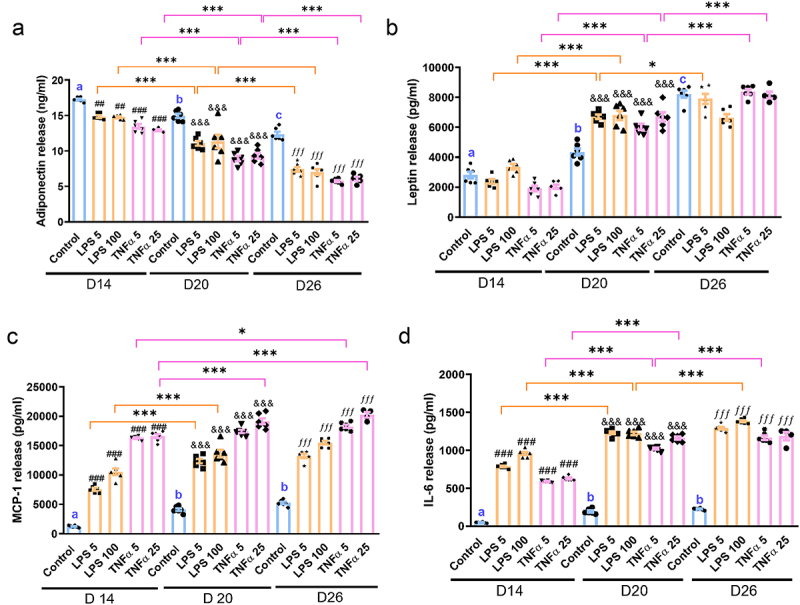
Effects of LPS and TNFα on adiponectin, leptin, MCP-1 and IL-6 release from human primary adipocytes at post-differentiation D14, D20 and D26. Human primary preadipocytes at post differentiation day 12, day 18 and day 24 were incubated with TNFα (5 or 25 ng/ml) for 48 h. Levels of adiponectin, leptin, MCP-1 and IL-6 release into cell culture medium were determined by ELISA. Results are expressed as means ± SEM (bars) for groups of 6. *p < .05, **p < .001. a vs b represents p < 0.05; b vs c represents p < 0.05, b vs b represents p > 0.05. ^##^*p* < 0.01, ^###^*p* < 0.001 compared to control (D14), ^&&&^*p* < 0.001 compared to control (D20), ^*fff*^*p* < 0.001 compared to control (D26).

Given the association between obesity and a state of low-grade chronic inflammation, we sought to investigate whether hypertrophic adipocytes contribute to a greater production of pro-inflammatory cytokines. To this end, we exposed human adipocytes at D12, D18, and D24 to LPS (5 ng/ml and 100 ng/ml) or TNFα (5 ng/ml and 25 ng/ml) for 48 h. As shown in Figure 3a, there was a decline in the secretion of the anti-inflammatory adipokine adiponectin as adipocytes become hypertrophy (D14 Control vs, D20 Control, *p* < 0.01; D20 Control vs D26 Control, *p* < 0.001). Conversely, the pro-inflammatory adipokine leptin, as well as the cytokines MCP1 and IL-6, exhibited an increase with adipocyte hypertrophy (Figure 3b–d). Furthermore, both LPS and TNFα intensified the suppression of adiponectin secretion in hypertrophic adipocytes (Figure 3a), while promoting an elevated release of leptin at D20 and D26 (Figure 3b). The release of MCP-1 and IL-6 was also significantly increased by LPS and TNFα at D14, with a further increase observed at D20 and D26 (Figure 3c–d). Consistently, we also observed an increase in the mRNA levels of IL-6 and MCP-1 in adipocytes that were treated with LPS or TNFα for 48 h, despite the induction of these mRNA levels were less prominent than their corresponding release level (Fig S2). In addition, there was no marked increase of LDH release between each treatment compared to the controls, indicating that LPS or TNFα did not exert toxicity in adipocytes (Fig S1).

### 3.4. Impaired intracellular inflammatory signalling pathways in human hypertrophic adipocytes

**Figure 4.**
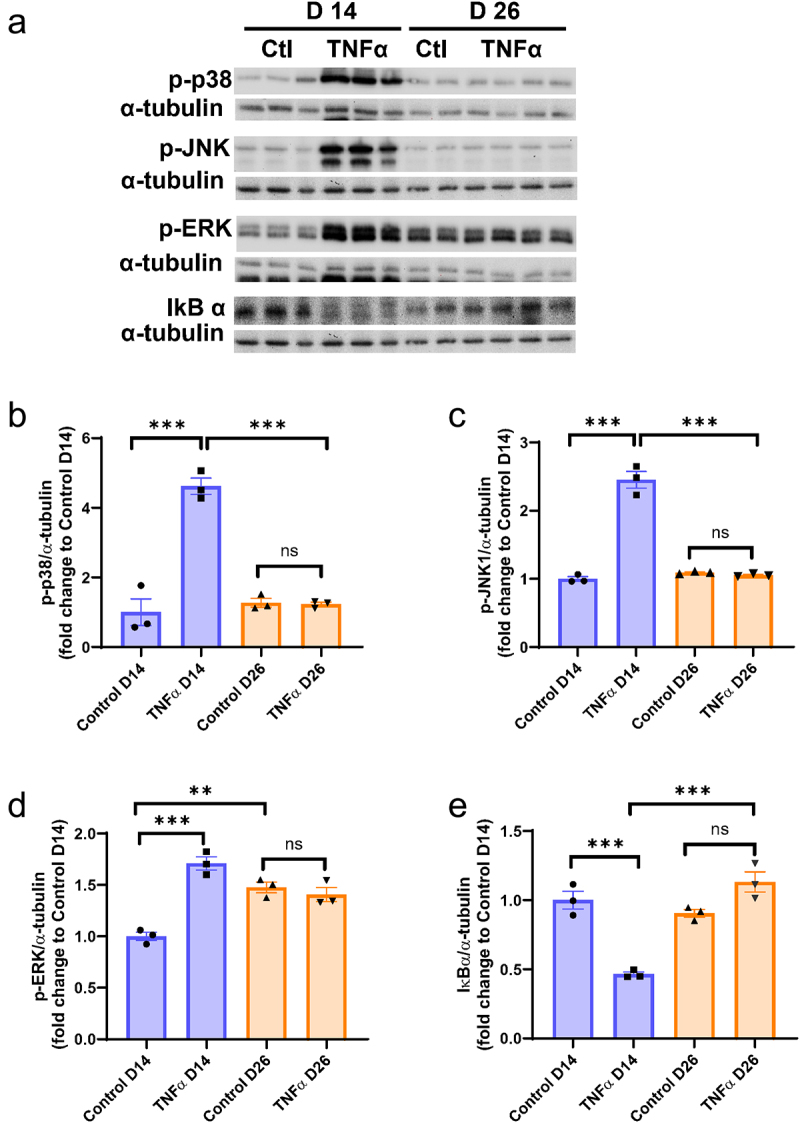
Effects of TNFα on MAPK activation and IκBα expression in human primary adipocytes at post differentiation day 14 and day 26. Adipocytes at day 14 and day 26 were incubated with TNFα (5 ng/ml) for 20 min; protein expression of phosphorylated p38, JNK, ERK and IκBα in cell lysates was analysed by Western blotting (a). The proteins expressions were quantified and normalized to α-tubulin levels and expressed as fold change to control (b-e). Data are means ± SEM (bars) for groups of 3. *p < .01, **p < .001.

To investigate the intracellular inflammatory signalling pathways following TNFα treatment, human mature adipocytes (D14) and hypertrophic adipocytes (D26) were stimulated with TNFα (5 ng/ml) for 20 minutes, and the activation of MAPK and IκBα expression was assessed. The results showed that there was a notable activation of p38, ERK, and JNK in adipocytes at D14 following acute TNFα treatment, whereas no activation was observed in adipocytes at D26 (Figure 4a–c). Additionally, there was a higher basal ERK activation in adipocytes at D26 compared to D14 (Figure 4d). Consistent with the activation of MAPK, the activation of NF-κB, as shown by the degradation of IκBα, was clearly observed in TNFα-treated D14 adipocytes. However, there was no impact on hypertrophic adipocytes (D26) (Figure 4e).

## 4. Discussion

In this study, we examined the secretion dynamics of adipokines and cytokines in human adipocytes over a 30-day culture period, mimicking the hypertrophy of human adipocytes. Our study revealed significant alterations in adipokine secretion profiles, inflammatory responses and intracellular signalling pathways in hypertrophic adipocytes compared to mature adipocytes, providing intricate mechanisms underlying human adipocyte dysfunctions in the context of obesity and obesity-related metabolic disorders.

Adipocyte hypertrophy has been indicated as an important factor contributing to the altered adipocyte function and metabolic complications such as type 2 diabetes [12] and non-alcoholic fatty liver disease [13]. In our study, human adipocyte hypertrophy was characterized by enlarged intracellular lipid droplets with increased lipolysis at D15 and afterwards. The significant increase in triglyceride content and heightened lipolysis observed in hypertrophic adipocytes are indicative of a shift in lipid metabolism. These findings align with the evidence from a chronological culture of mouse 3T3-L1 adipocytes [8] and an *in vitro* human adipocyte spheroids hypertrophied model [14]. Previous studies have utilized mature adipocytes treated with fatty acids [15] or glucose [16] as *in vitro* models of adipocyte hypertrophy. However, it remains challenging to discern whether the observed alterations in adipocyte function are primarily attributable to the effects of fatty acids and glucose or specifically to the hypertrophic state of the adipocytes.

One of the key observations was the unbalanced adipokine secretion in hypertrophic human adipocytes compared to mature adipocyte under both basal and stimulated conditions. The secretion of anti-inflammatory adipokine adiponectin is restricted to adipocytes and reaches its highest level in mature adipocytes (day 9) and then decreases in hypertrophic adipocytes till day 30. Adiponectin, an effective insulin sensitizer, demonstrates robust anti-inflammatory characteristics through its regulatory role in macrophage polarizations within adipose tissue [17]. Furthermore, the susceptibility of hypertrophic adipocytes to inflammatory stimuli is underscored by the exacerbation of adiponectin reduction induced by both LPS and TNFα. This finding aligns with a prior investigation which shown that proinflammatory cytokines decrease the production of adiponectin in 3T3-L1 adipocytes [18]. In contrast, the release of pro-inflammatory adipokine leptin peaked on day 15 and remains elevated afterwards, suggesting a sustained higher levels of leptin in human adipocytes with hypertrophy. Indeed, human obesity is linked to elevated circulating levels of leptin [19] and our findings indicate that adipocyte hypertrophy may play a significant role in contributing to the hyperleptinemia observed in individuals with obesity. Moreover, in line with a prior study [20], human preadipocytes exhibited significantly elevated levels of pro-inflammatory cytokine production, including IL-6, IL-8, and MCP1, in comparison to differentiated adipocytes. LPS and TNFα induces higher levels of MCP1 and IL-6 release in mature human adipocytes (D14), there was further increase of these pro-inflammatory cytokines release in hypertrophic adipocytes (D20 and D26), suggesting that human adipocytes exhibit a progressive amplified inflammatory state as adipocytes undergo hypertrophy. Furthermore, our study revealed disrupted intracellular inflammatory signalling pathways in human hypertrophic adipocytes following acute TNFα stimulation. This was marked by compromised activation of p38 and JNK MAPK, along with sustained activation of ERK and IκBα, indicating a gradual reduction in signalling effectiveness with adipocyte hypertrophy. The NF-κB pathway has been shown to play a pivotal role in adipose inflammation and subsequent insulin resistance [21]. NF-κB signalling activation enhances the production of TNF-α, IL-6, and MCP-1, resulting in the phosphorylation of IRS-1 at serine residues and impairs insulin signalling [22]. The p38 and JNK MAPK signalling pathways were found to be significantly associated with inflammation in obesity, as well as to be contributors to insulin resistance and metabolic disorders induced by obesity. JNK signalling is activated in adipocytes in obese humans and mice, which further activate the transcriptional regulator AP-1 and induces the expression of inflammatory genes, such as IL-6 and TNFα, inhibiting insulin signalling and causing insulin resistance [23]. The p38 pathway was initially described as a master regulator of pro-inflammatory cytokine secretion in macrophages [24]. In adipocytes, the p38 pathway has been reported plays a regulatory role in adipose tissue secretome, as inhibiting p38 leads to a reduction in the secretion of TNF-α-induced IL-6 and leptin [25,26]. Nonetheless, the observed impairment in the activation of p38, JNK, and NF-κB signalling pathways in human hypertrophic adipocytes strongly implies a significant functional defect in these cells and may contribute to the sustained low-grade inflammation observed in obesity and the associated metabolic dysfunctions. Gaining insight into the processes behind these signalling abnormalities in hypertrophic adipocytes may offer promising avenues for therapeutic interventions to restore adipocyte function and alleviating the metabolic complications associated with obesity.

In conclusion, our study provides a comprehensive analysis of human hypertrophic adipocytes, encompassing lipid metabolism, adipokine secretion profiles under both basal and inflammatory stimulated conditions, and the intracellular signalling pathways involved. Our results offer valuable insights into the dysregulated adipokine secretion and inflammatory responses in human adipocyte hypertrophy, highlighting the critical roles of adipokines and inflammatory pathways in the development of adipocyte dysfunction in obesity and related metabolic disorders.

## Supplementary Material

Supplemental Material

## Data Availability

All data generated or analysed during this study have been deposited in the ScienceDB database under Doi number: 10.57760/sciencedb.08997, link: https://www.scidb.cn/en/s/EFbIJz
