# Supplementary material for: Disrupted adipokine secretion and inflammatory responses in human adipocyte hypertrophy
Source: Adipocyte. 2025 Apr 3;14(1):2485927. doi: 10.1080/21623945.2025.2485927 (PMC11980453; doi:10.1080/21623945.2025.2485927)
Supplement: Supplemental Material [file KADI_A_2485927_SM2775.docx]

s
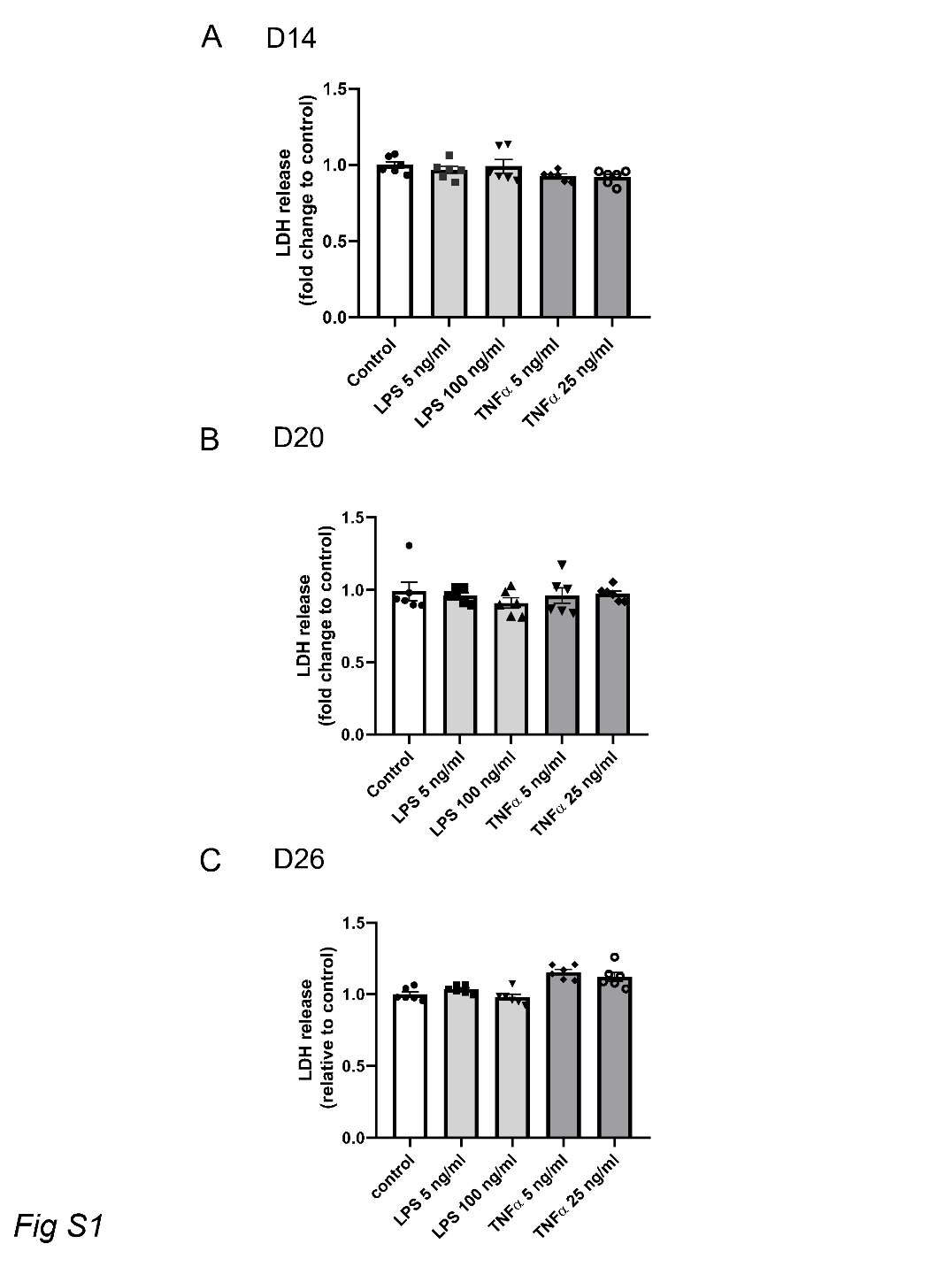


**Figure S1.** Cell viability of adipocyte treated with TNFα and LPS. Human primary adipocytes at post-differentiation D12, D18 and D24 were incubated with TNFα (5 and 25 ng/ml) or LPS (5 and 100 ng/ml) for 48 h. Cell viability were determined by LDH release assay. Data are means ± SEM (bars) for groups of 6.


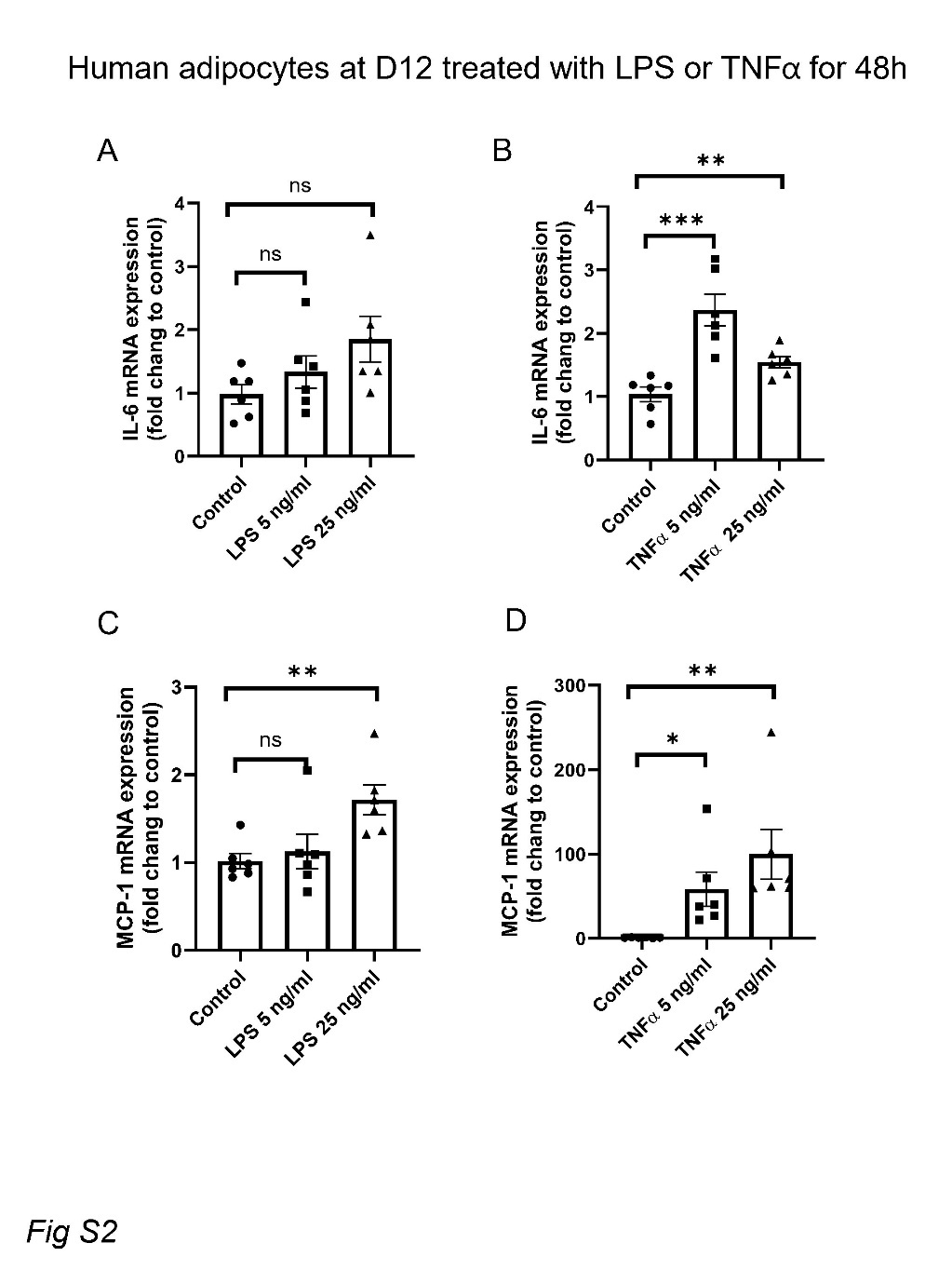


**Figure S2.** Effects of LPS or TNFα on inflammatory cytokine mRNA expressions. Human primary adipocytes at D12 treated with LPS (5ng/ml and 25 ng/ml) or TNFα (5 ng/ml and 25 ng/ml) for 48h. mRNA levels of pro-inflammatory cytokine IL-6 and MCP-1 were determined by real-time qRT-PCR and calculated as fold change to control. Data are means ± SEM (bars) for groups of 6. *P<0.0, **P<0.01, ***P<0.001.


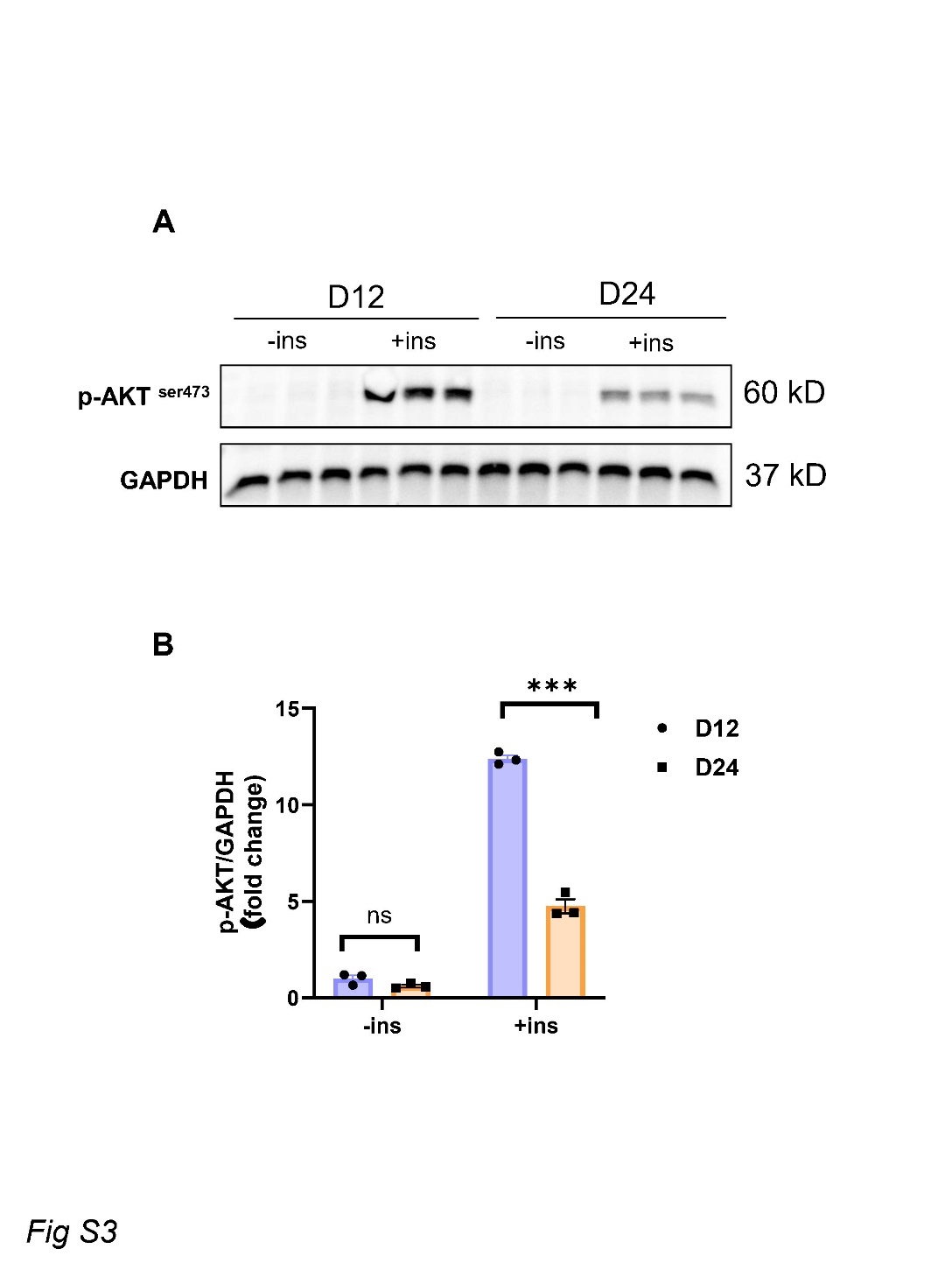


**Figure S3.** Insulin signaling in human primary adipocytes at post-differentiation day 12 and day 24. Adipocytes were incubated in DMEM/F12 without FBS for 24 h before being stimulated with insulin (167 nmol/l) for 5 min; Akt phosphorylation at Ser473 (pAkt) was analyzed by Western blotting and densitometry. GAPDH were used as loading controls. Data are means ± SEM (bars) for groups of 6. ***P<0.001.
